# Prediction of fluid responsiveness in mechanically ventilated children using dynamic and static parameters by esophageal Doppler in a pediatric ICU

**DOI:** 10.1186/cc14262

**Published:** 2015-03-16

**Authors:** K El Halimi, M Negadi, H Bouguetof, L Zemour, D Boumendil, Z Chentouf Mentouri

**Affiliations:** 1 University Ahmed Benbella Oran 1, Oran, Algeria

## Introduction

Prediction of fluid responsiveness is defined by an increase in stroke volume (SV) of at least 10% after volume expansion. Dynamic [1] and static [2] esophageal Doppler (OD) parameters have been proposed in mechanically ventilated children to guide fluid therapy. This study aimed to compare dynamic parameters using the respiratory variation in aortic blood flow with static parameters using Doppler corrected flow times (FTc) obtained by OD.

## Methods

A prospective, observational and interventional study was conducted in our pediatric ICU from March 2012 to September 2014. We investigated 18 mechanically ventilated children with acute circulatory failure (ACF) - tachycardia, hypotension, oliguria, delayed capillary refilling or hemodynamic instability despite vasopressor drugs - using OD for each patient. Intervention: standardized volume expansion (VE).

## Results

The VE-induced increase in stroke volume was ≥10% in 14 patients (responders) and <10% in four patients (nonresponders). Before VE, the DELTA Vpeak ao in responders was higher than in nonresponders (19.5% (12 to 29) vs. 11.5% (7 to 13)), whereas FTc was lower in responders than in nonresponders (262.5 milliseconds (180 to 340) vs. 285 milliseconds (205 to 300)). The prediction of fluid responsiveness was higher with DELTA Vpeak ao (ROC curve area 0.964 (95% CI = 0.756 to 1.000); *P *= 0.0001) than with FTc (ROC curve area 0.562 (95% CI = 0.314 to 0.790); *P *= 0.7203). The best cutoff value for DELTA Vpeak ao was 13% with sensitivity and specificity predictive values of 85.7% and 100%, respectively; and the best cutoff value for FTc was 265 milliseconds with sensitivity and specificity predictive values of 57.1% and 75%, respectively.

## Conclusion

In our study, DELTA Vpeak was the most appropriate variable to predict fluid responsiveness by OD in ventilated children with ACF.
